# Sex differences in Parkinson's Disease: An emerging health question

**DOI:** 10.1016/j.clinsp.2022.100121

**Published:** 2022-10-01

**Authors:** Luiz Philipe de Souza Ferreira, Rafael André da Silva, Matheus Marques Mesquita da Costa, Vinicius Moraes de Paiva Roda, Santiago Vizcaino, Nilma R.L.L. Janisset, Renata Ramos Vieira, José Marcos Sanches, José Maria Soares Junior, Manuel de Jesus Simões

**Affiliations:** a Structural and Functional Biology Graduate Program, Escola Paulista de Medicina, Universidade Federal de São Paulo (EPM/UNIFESP), São Paulo, SP, Brazil; b Biosciences Graduate Program, Instituto de Biociências, Letras e Ciências Exatas, Universidade Estadual Paulista (IBILCE/UNESP), São José do Rio Preto, SP, Brazil; c Medical Sciences Graduate Program, Universidade Federal do Ceará (UFC), Fortaleza, CE, Brazil; d Life Systems Biology Graduate Program, Instituto de Ciências Biomédicas, Universidade de São Paulo (ICB/USP), São Paulo, SP, Brazil; e Department of Ophthalmology, University of Texas Southwestern Medical Center, Dallas, United States; f Department of Pharmacology, Escola Paulista de Medicina, Universidade Federal de São Paulo (EPM/UNIFESP), São Paulo, SP, Brazil; g Department of Obstetrics and Gynecology, Faculdade de Medicina, Universidade de São Paulo (FMUSP), São Paulo, SP, Brazil

Parkinson's disease (PD) is a neurodegenerative disease of high incidence in the global population, affecting 10 million people.^1^^,^^2^ In Brazil, it is estimated that in 2030 more than 600 thousand people will develop the disease.^3^ PD occurs in both sexes (male and female), with 1% of men and women developing it at the age of 45–54 years and subsequently 4% of men and 2% of women at the age of 85 years.^1^^,^^2^ PD is a debilitating pathology for the patient, in addition, it generates a large financial burden on governments worldwide with social security expenses, medications, loss of income, etc, generating a cost of about 52 billion USD specifically in the United States.^2^

Currently, neuroscientists discuss the importance of biological sex as a significant factor related to the severity of PD.^4^ Since 2015, the importance of biological sex has been highlighted by the National Institutes of Health (NIH) as a crucial variable for rigorous research (NOT-OD-15-102). Even though it is imperative that clinical and basic studies include sex in investigations, few studies consider researching the variable as an objective for the investigation.^5^, ^6^, ^7^

The sex difference is a pivotal aspect that defines the epidemiological and clinical implications of PD.^8^, ^9^, ^10^ Although the prevalence of PD is double in men than in women,^9^^,^^10^ the disease's progression and mortality rates are higher in women.^11^^,^^12^ Regarding the most common PD symptoms, motor symptoms help characterize and diagnose PD, where women and men show significant differences in clinical progression and treatments.^13^ Although previous work could not relate sex differences in PD, important data has been published that women, in comparison to men, present later onset of motor symptoms,^14^ tremor-dominant,^15^ and higher striatal dopaminergic activity.^15^^,^^16^ Other clinical aspects such as cognition, emotional and mental impairments are key clues for PD manifestation.^10^ Studies hve shown that cognitive impairment and dementia are significantly more prominent in men with PD.^14^^,^^15^

Sensory impairments in taste and smell are more common in men compared to women.^16^ Depressive symptoms such as irritability and agitation, loss of pleasure self-dislike, self-punishment, and feelings of worthlessness are more prominent in women with PD.^17^^,^^18^, ^19^, ^20^ In addition, women showed earlier dyskinesias and worst postural instability.^21^^,^^17^ On the other hand, men with PD are more likely to present with writing difficulties, clumsiness,^22^ higher bradykinesia and rigidity scores compared to women with PD.^21^^,^^17^^,^^23^

In addition to motor findings, sleep disorders are also different between the sexes. In female patients, insomnia is a recurrent disorder,^24^ where women are more prone to the development of symptoms such as fatigue, pain, neuropsychiatric disorders (anxiety and depression), restless legs syndrome, and constipation. Such symptoms can disturb the cycles of sleep, in contrast, men are more resistant to sleeping disorders, which could explain the higher prevalence of insomnia in the female sex.^25^ REM sleep behavior disorders (RBD) are evidenced in both sexes in patients with PD. Severity of symptoms may vary depending on the sex,^26^ female PD patients reported significantly fewer fights and aggressive behavior during dreams and had more disturbed sleep.^25^

The pharmacology of PD is varied, although there is no availability of drugs that inhibit the progression of neurodegeneration, there are several types of pharmacological agents used for the treatment of PD symptoms. Among them, the authors can highlight levodopa (LD), dopamine agonists, and catechol-Omethyltransferase inhibitor (COMT), and monoamine oxidase type B inhibitors (MAO-B).^27^ During the disease, the pharmacological prescription depends on different factors, such as age, motor, and non-motor symptoms, however, it is still not common to take into consideration the sex of the individual.^28^^,^^29^

LD in addition to dopamine decarboxylase inhibitors (e.g. carbidopa) is considered the most effective pharmacological option for the treatment of motor symptoms, however, the treatment can be initiated with other drugs, such as dopamine agonists (Pramipexole and Ropinirole) or anticholinergics (Benztropine), to decrease complications related to the prolonged use of LD.^27^^,^^30^ Once treatment with LD is initiated, adjunctive therapy with COMT (Entacapone and Tolcapone) and MAO-B inhibitors (Selegiline and Rasagiline) or dopamine agonists to manage motor fluctuations is common.^30^

Evidence shows that there are sex differences in pharmacokinetics and pharmacodynamics during the administration of PD drugs. Tolcapone is a COMT inhibitor used as an adjuvant with LD, although well tolerated, females are more susceptible to gastrointestinal and orthostatic adverse effects.^31^ Pramipexole, a dopaminergic agonist, presents a higher bioavailability in females, probably related to a lower oral clearance.^32^ It was also demonstrated, in the female sex, a higher LD bioavailability, when compared to the male sex, evidenced by a higher AUC and Cmax, measures used to demonstrate the drug plasma concentration.^33^^,^^34^^,^^35^

Body weight can be a factor to explain sex differences since the results point out that the female sex is correlated with lower body weight. However, even after adjusting for this factor, the difference remains, indicating that there are other mechanisms, besides body weight, which is involved in the higher levodopa availability in females.^33^^,^^35^^,^^36^ In part, this difference may be associated with lower LD clearance^37^ and COMT activity in females,^38^ in addition to 17β-estradiol inducing a decrease in COMT expression.^39^ More studies are needed to clarify the issue that sex can alter the response to drugs.

This proves to be relevant because female subjects are more likely to develop levodopa-induced dyskinesia, one of the main complications associated with the treatment.^40^ Moreover, during the DEEP study, it was observed that the female gender is associated with an 80.1% higher risk of developing wearing-off (WO), a term which refers to the decrease in the time in which LD controls the symptoms.^41^ During the "off" periods it is common for patients to experience a return of motor and non-motor symptoms, significant being that females have a higher risk of WO.^41^ Impulse control disorders, although present in the PD population in general, may be associated with the intake of high doses of LD and dopaminergic agonist.^42^ The male gender can be considered a risk factor for addiction to gambling (pathological gambling) and hypersexuality^43^ and is associated with punding and compulsive drug use.^44^

Differences are also found in the neurodegenerative process. The cholinergic dopaminergic denervation of the caudate and neocortical nucleus are higher in male patients than compared in females.^12^ Women showed higher amounts of striatal dopamine when compared to men,^45^ and higher levels of the striatal dopamine transporter, these findings suggest higher protection for the development of PD linked in males.^46^

A higher expression of glutamate and vesicular glutamate transporter 2 (VGLLUT2) in dopaminergic (DA) neurons has been shown in women compared to males, signifying higher protection of women to DA neuron loss and a higher permeability linked to males.^47^ Genes involved in PD pathogenesis are α -synuclein and PINK1 which are under-expressed in males, on the other hand, signal transduction and neuronal maturation regulatory genes are positively regulated in females.^48^ These findings reveal important genetic characteristics since DA neurons in the substantia nigra are sensitive to stress conditions, and the positive regulation of genes such as α -synuclein and PINK1 a protein required for mitophagy in DA neurons.^49^ These findings may interfere with treatment strategies and motor or non-motor symptoms in PD.

Since there are many differences found between the sexes, a neuroprotective effect may be implicated, suggesting that there may be an endogenous hormonal role. Times of hormonal instability, such as menopause, might have a role in the progression of PD,^50^ but some studies have not found an association between PD and fertile lifespan, age of menarche, or the age of menopause.^51^ Hormonal therapy has been suggested as a potential treatment of PD. Estrogen has been found to be neuroprotective and may protect neurons from toxins.^52^ Due to increased estrogen levels, the endometrium may grow and cause endometrial hyperplasia- enlargement of the uterine wall.

Progestin, a synthetic form of progesterone, is used to prevent this growth and therefore must be taken along with the therapy.^53^ However, the use of estrogen and progestin hormonal therapy may increase the risk of PD in some studies. In contrast, Gatto et al and collaborators^54^ showed that having either endogenous or exogenous estrogen therapy for the longest cumulative time had reduced PD risk. Conjugated equine estrogen hormonal therapy seems to ameliorate the motor symptoms of PD after it has been diagnosed in postmenopausal women.^55^

Men's decline in testosterone during aging and might have a role in the progression of PD.^56^ Despite the drop in testosterone, studies show that using the androgen (testosterone) hormonal therapy does not seem to have similar neuroprotective effects as found in estrogen.^57^ More studies are needed to understand if supplementation of testosterone creates a risk factor for PD progression and/or alleviates symptoms of PD.

These are just the more common of the various sex hormones that are avaiable to the two sexes. More research is needed to describe the nuances of sex hormones relating to the onset and progression of PD. Also, during development, sex hormones like testosterone can make the body sexually dimorphic, such as the substantia nigra the brain area important for PD pathology are dimorphic in mice.^58^ The study of sex hormones in the progression neurodegenerative diseases will be a challenging and rewarding area to research.

Finally, the lack of studies on humans that report the difference of sex in clinical features, symptoms, treatment, neurodegeneration and hormones exposes new avenues of research. Therefore, the authors must consider these issues and we hope that future studies can obtain more information about these differences to circumvent the lack of personalized treatments for men and women affected by PD. Above all, the authors emphasize in this editorial that the differences in the sex response profile of treatments should be an area of interest.

In conclusion, it is interesting to take into consideration the disparity that exists between the sexes. Especially in the pharmacological dosage, since different responses, tolerance, and pharmacokinetics exist. Moreover, special attention to this issue could allow better management of adverse effects that often cause limitations to pharmacological therapy, such as WO and the emergence of dyskinesias.

## CRediT authorship contribution statement

**Luiz Philipe de Souza Ferreira:** Conceptualization, Investigation, Visualization, Writing – original draft. **Rafael André da Silva:** Conceptualization, Investigation, Visualization, Writing – original draft. **Matheus Marques Mesquita da Costa:** Conceptualization, Investigation, Visualization, Writing – original draft. **Vinicius Moraes de Paiva Roda:** Conceptualization, Investigation, Visualization, Writing – original draft. **Santiago Vizcaino:** Conceptualization, Investigation, Visualization, Writing – original draft. **Nilma R.L.L. Janisset:** Writing – review & editing. **Renata Ramos Vieira:** Writing – review & editing. **José Marcos Sanches:** Supervision, Writing – original draft, Writing – review & editing. **José Maria Soares Junior:** Supervision, Writing – original draft, Writing – review & editing. **Manuel de Jesus Simões:** Supervision, Writing – original draft, Writing – review & editing.

## Declaration of Competing Interest

The authors declare no conflict of interest. The funders had no role in the design of the study; in the writing of the manuscript, or in the decision to publish.
